# Pleurodynia

**Published:** 1888-02

**Authors:** R. P. Davies

**Affiliations:** Petty, Texas


### PLEURODYNIA.

*By R. P. Davies, M. D., Petty, Texas.*

For Daniel's Texas Medical Journal.

MY REASONS for contributing this article to your JOURNAL and for its many readers are, that from what I have read in several text books and heard from many physicians, the above diseased condition, or Pleurodynia, as I think best to term it, is very little understood, and as poorly treated; and again, it is very often, and I might say, most generally called by some other name or names, as, for instance, in Naphey's Medical Therapeutics it is called an intercostal neuralgia. In Reynolds' System of Medicine it is called an intercostal rheumatism, or pleurodynia, and very justly I think. In Thomas' Dictionary it is defined as intercostal rheumatism. So much for three leading text books on medicine Among some of my neighboring physicians it is called Pleurisy; others have called it Angina Pectoris; others still call it Pneumonia; especially if there is any bronchial, laryngeal, or any of the common cough or colds or symptoms attending it.

But enough for the name. I will now endeavor to describe several cases of that disease, with my plan of treatment. Among my patients during the present weather, and especially since the very cold and damp weather of the past six or seven weeks, there has occurred eight cases of Pleurodynia, or intercostal rheumatism, which ever you wish to call it. A history of a single case with all the common symptoms will suffice for all eight, as the symptoms of one were present in all, with very few unimportant exceptions. In all these eight cases I was called very hurriedly and urgently.

On arriving at the patients' place I invariably found them in a sitting posture complaining of great pains in and around the heart, or in close proximity to the heart, with almost an inability to get a good breath. The patient would tell me between gasps that if I did not relieve him soon that this could not last much longer, and that they believed they had heart disease or asthma; their respiration was short and shallow, countenance bleached or sallow, eyes protruded and dark circles around them, no fever in a single case; heart movements a little accelerated, with some pulse excitement; no cough or soreness of throat, no dizziness, or coating on tongue; some chilliness or coldness of skin; bowels and kidneys rather obstructed, and especially the bowels; in most of the patients the bowels had not acted for several days, the kidneys had acted very well in all, but urine was high colored and burning in passing; appetite various, sometimes voracious and at other times none, but all could eat some at their regular meal times. On getting a previous history of their cases, all described among the first symptoms a condition of cervicodynia, with now and then lumbodynia, or both. For a day or two preceding the attack of pleurodynia the patients said that the pains of the cervical and lumbar regions had left these situations, and all had gathered or settled in the breast and sides, and that these pains had so changed during the night, while asleep. At any rate, most all the cases were attacked during the night after a few hours of sleep, Some had arisen from the bed in order to procure more bed clothing, and would be taken with a rigor, and then the pleurodynia would first show itself. Others were awakened by the very severe pains in the chest and shortness of breath.

As stated above, all of the patients suffered the most excruciating pains in some locality of the chest walls, most generally in and around the region of the heart and diaphragm. All had shortness of breath, or a suffocating sensation and a feeling of impending death. In the case of one of my patients who had been under another physicians' treatment, the physician told the friends of the patient that it was a case of rheumatism of the heart, and that no doubt the patient would die soon. Another physician informed the husband of a second patient that she "had Angina Pectoris, and that that disease had never been cured; it was that disease which so suddenly killed Professor L. P. Yandell, of Louisville, Ky." A third physician told another case that he had dropsy of the chest, and that in all probability it would kill the patient very soon.

All the above mentioned cases, with five others, came under my treatment and are at this time enjoying the best of health, and all will no doubt live out their allotted time. My treatment in all these cases was that of purgation, diuretics and diaphoretics, with counter irritation. Anodynes or treatment for pains did no good for the time being. Neither did I use any quinine or digitalis, nor aconite. But my first prescription was:

| | |
|---|---|
| Calomel............................................ | 30 grs |
| Aloine.............................................. | 6 " |
| Soda Bicarbonate.................................. | 15 " |

Divided into three powders and one given every three hours. Should the powders fail to act well and properly within two hours after giving the last one, I would give a tablespoonful of Epsom salts or castor oil; then, as soon as the purgative medicine had cleaned out the stomach and bowels I prescribed the following mixture:

| | |
|---|---|
| Potass Bicarbonate | ℥ i |
| " Nitras | ʒ ii |
| Guaiac Tinct. | ℥ ii |
| Colchicum wine | ʒ ii |
| Buchu Fld. Ext. | ℥ ii |

Mix, and give a tablespoonful in a wine glass of sweetened water every three hours.

For a counter irritant I invariably used a plaster of the good old Spanish fly blistering ointment, a plaster about four by six inches in size, applied right between the shoulder blades, just below the nape of the neck, and allowed to remain on that location three hours; the plaster was then taken off and an oiled cloth put over the site of the plaster, and there kept until blisters arose, which would occur in three hours, the blisters opened and dressed as usual. Just as soon as the plaster would blister the patients would become free from pains, and a general improvement would begin at once. I made the patients continue taking the potash or alkaline mixture until all was taken, and then I put them on a tonic like this:

| | |
|---|---|
| Sarsaparilla Syr. Comp. | 4℥ |
| Iron Syr. Iodide | 1 " |
| Potass Iodide | 1ʒ |
| Cinchona Tinct. Comp. | 4℥ |

A tablespoonful before each meal.

In connection with this article I hereby append an extract from a letter received yesterday from a very highly esteemed pupil and friend, Dr. A. J. Rush, of Delta county, Texas. In the letter among the reports of several cases that he had very recently had under treatment he mentions one that had been under another physicians' treatment. He thus writes:

"I also took another case from the same doctor, of heart disease. The man got up at night to get some more cover for his bed, and took a slight rigor, followed by severe pain around the heart. It was six miles to me and only two miles to the other doctor, so they sent for the nearest doctor, the nearest doctor pronouncing it neuralgia or angina pectoris, and after two or three days of treatment from the near doctor without any relief, I was sent for. Found heart very much excited; apex beat directly under left nipple. There was a little abnormal sound just prior to systolic contraction which I found afterwards to be due to the accelerated action of the heart. He had soreness all around the diaphragmatic attachments; had smothering spells and very excruciating pains in the region of the heart. So the best I could make out was a rheumatic condition of the heart. After detailing the treatment and some other unimportant symptoms Dr. Rush asked me to diagnose the case. I have done so, and call it pleurodynia, and now, to you, Mr. Editor, and your many readers, I leave the above article for all it is worth, and for what it is worth, at the same time, wishing you all a happy and successful new year.

